# Supplementary material for: CcMYB12 Positively Regulates Flavonoid Accumulation during Fruit Development in Carya cathayensis and Has a Role in Abiotic Stress Responses
Source: Int J Mol Sci. 2022 Dec 9;23(24):15618. doi: 10.3390/ijms232415618 (PMC9779359; doi:10.3390/ijms232415618)
Supplement: Supplementary file 1 [file ijms-23-15618-s001.zip › Table S1.pdf]

Table S1

S1	CCA0705S0001	CCA0634S0058	CCA1196S0013	CCA0779S0067	CCA0816S0015			
S2	CCA1289S0001	CCA0757S0066	CCA0912S0003	CCA0510S0131				
S3	CCA0563S0066							
S4	CCA0638S0185	CCA0748S0009	CCA0522S0037	CCA0816S0089	CCA0779S0016			
S5	CCA1382S0024							
S6	CCA0887S0030	CCA0887S0029						
S7	CCA0526S0313	CCA0691S0036	CCA1161S0009	CCA1381S0001	CCA0857S0055	CCA1410S0053	CCA1236S0030	CCA1161S0008
S9	CCA0779S0047	CCA0816S0043	CCA1309S0008	CCA1309S0003	CCA0912S0044	CCA0912S0054	CCA0500S0004	
S10	CCA0954S0020	CCA1724S0002	CCA1046S0036	CCA1055S0070				
S11	CCA0961S0018	CCA0576S0060	CCA0568S0059	CCA0628S0078	CCA1603S0008			
S12	CCA0632S0174	CCA0789S0045	CCA0800S0132	CCA1126S0077				
S13	CCA0791S0008	CCA1111S0034	CCA0633S0098					
S14	CCA0779S0112	CCA0516S0022	CCA1073S0035	CCA1057S0050	CCA0704S0070	CCA1606S0014	CCA0834S0049	CCA0578S0020
	CCA0007S0005	CCA0879S0071	CCA0533S0220	CCA1097S0043	CCA1430S0009	CCA0680S0023		
S15	CCA1168S0035	CCA1132S0027	CCA1788S0002	CCA0633S0045				
S16	CCA0800S0131							
S18	CCA0616S0013	CCA0082S0007	CCA0799S0040	CCA1013S0028	CCA0552S0187	CCA0826S0075		
S19	CCA1094S0019							
S20	CCA0526S0256	CCA1057S0032	CCA0737S0065	CCA0762S0050	CCA1045S0020	CCA0922S0013	CCA0984S0066	
S21	CCA0605S0052	CCA1147S0056	CCA0573S0003	CCA1126S0086	CCA0883S0023	CCA0819S0012	CCA0884S0122	
S22	CCA0774S0069	CCA0525S0079	CCA0929S0034	CCA0670S0116	CCA1550S0010	CCA0912S0106		
S23	CCA0784S0147	CCA0784S0053						
S24	CCA0001S0007	CCA1217S0050	CCA0514S0238	CCA0656S0018				
S25	CCA1244S0016	CCA1157S0021	CCA0524S0280	CCA0582S0107	CCA0779S0170	CCA1094S0010		
