# Supplementary material for: CcMYB12 Positively Regulates Flavonoid Accumulation during Fruit Development in Carya cathayensis and Has a Role in Abiotic Stress Responses
Source: Int J Mol Sci. 2022 Dec 9;23(24):15618. doi: 10.3390/ijms232415618 (PMC9779359; doi:10.3390/ijms232415618)
Supplement: Supplementary file 1 [file ijms-23-15618-s001.zip › Table S2.pdf]

Table S2

Genes	Forward Primer (5' –3′)	Reverse Primer (5' –3′)
**Subcellular Localization Assay**		
CcMYB12	gagctcggtacccggggatccATGGGAAGGGCACCATGTTG	catgtcgactctagaggatccTTTTTCTTCAGTACTACCTTTGTTGCT
**Dual-luciferase reporter (DLR) assay**		
CcMYB12	cgctctagaactagtggatccATGGGAAGGGCACCATGTTG	ttcctgcagcccgggggatccCTATTTTTCTTCAGTACTACCTTTGTTGC
CcC4H	ttcctgcagcccgggggatccGTTCTAAATAGAGAAACTTCAAGTAGGCTT	cgctctagaactagtggatccTTTCGTGATGAAGAAAGAAGGGA
CcCHS	ttcctgcagcccgggggatccAAAAATCAGGTAGAGCATTGGATTG	cgctctagaactagtggatccGCCTCTTGCTCGACCCTAGTT
CcCHI	ttcctgcagcccgggggatccATGCACTTTTCAGGGGGAGTAA	cgctctagaactagtggatccTTTGACGCGTATATTAGTGGTAACTATG
CcF3H	ttcctgcagcccgggggatccATATATCAAATCACGTCAATTTGTGAGA	cgctctagaactagtggatccTTTGCAGGAACTGAATTGAGTTGC
CcF3'H	ttcctgcagcccgggggatccTTGATTTGTCGTGTTTTGAATTTTT	cgctctagaactagtggatccGGCGAGCTTTTTGTCTGTGC
CcF3'5'H	ttcctgcagcccgggggatccACTTCCATTTTTCAGATTTTACTTGAA	cgctctagaactagtggatccGGTGTTGCAGGCTCTTGCA
CcANS	ttcctgcagcccgggggatccAAAGATAAATACATTATGGTGTAACTTATATCG	cgctctagaactagtggatccTGTAAGAAAAGCAGCACGCTTT
CcANR	ttcctgcagcccgggggatccAGGACCTTTTTAAAGATTACTACTCATATTT	cgctctagaactagtggatccGGAGTTCGATATTCAGTGCTACCTT
CcDFR	ttcctgcagcccgggggatccTTTTTTTTCTTTCATATATAAAAGCGAA	cgctctagaactagtggatccGCTCCTTTTTCTTAATAAAAAGAAAACA
**Transactivation Analysis**		
CcMYB12	atggccatggaggccgaattcATGGGAAGGGCACCATGTTG	tcgacggatccccgggaattcCTATTTTTCTTCAGTACTACCTTTGTTGC
**Arabidopsis Transformation**		
CcMYB12	gagctcggtacccggggatccATGGGAAGGGCACCATGTTG	catgtcgactctagaggatccTTTTTCTTCAGTACTACCTTTGTTGCT
**qRT-PCR**		
CcMYB12	CGGGGAGTGTTACGATTGCT	TCCCATAAGGCCCATTGGTT
CcACTIN	GCGACAATGGAACTGGAA	CCTCTTTGACTGGGCTTC
CcPAL	GGCCAGGAAAGACCAGAAG	GACAAGACCATGACGATGAT
CcCHS	GTTGGGCTTACATTCCATCTC	GCTTCAGAGCCAACTTTTC
CcCHI	AAAGATGAGTCCATTCCCG	TGATTTTCGGCTTCTGTATC
CcF3H	ACCATCACTCTGTTGTTGC	CTTGAACCTCCCGTTACTC
CcF3'H	TCAAATCAATGGTGGTGGAG	AACGATGGCTGTCAAAAAC
CcF3'5'H	TCAAGCATAATAGAGTGGGC	AGGTTTAGTGGGGTAGACG
CcFLS	GCTCTCATTATTCACATCGG	GGAGGGTTCTCTTCACTAAC
CcFNS	GAGGTGGCGGACAAGCTATT	CAAGTGCTAGATCAGGGCGA
AtPAL	ATCGAAGTGATCCGTTACGC	ACTCCGATTGGTGTTCCTTG
AtC4H	TCGACACAGTTCTTGGACCG	TGGAGGTTCATGTGAGGCAC
At4CL	CGCAAACCCTTTCTTCACTC	ACTCCGTCGTCGTTTTGAAG
AtCHS	TGAGAACCATGTGCTTCAGG	CAGATGCATGTGACGTTTCC
AtF3H	TCAGATCGTTGAGGCTTGTG	ATGTCGAAACGGAGCTTGTC
AtF3'H	CCTCCACCTCCGACTAGGGT	TGCTCGGCCACGGATTTA
AtANS	TCAAGAAAGCCGGAGAAGAG	TTGTCCACTCGCGTTGTTAG
AtDFR	GTCGGTCCATTCATCACAAC	TGAGCGTTGCATAAGTCGTC
AtACTIN	TGCTGGATTCTGGTGATGGT	ATTTCCCGCTCTGCTGTG
